# Feature Importance of Stabilised Rammed Earth Components Affecting the Compressive Strength Calculated with Explainable Artificial Intelligence Tools

**DOI:** 10.3390/ma13102317

**Published:** 2020-05-18

**Authors:** Hubert Anysz, Łukasz Brzozowski, Wojciech Kretowicz, Piotr Narloch

**Affiliations:** 1 Faculty of Civil Engineering, Warsaw University of Technology, Al. Armii Ludowej 16, 00-637 Warsaw, Poland; p.narloch@il.pw.edu.pl; 2 Faculty of Mathematics and Information Science, Warsaw University of Technology, Koszykowa 75, 00-662 Warsaw, Poland; l.brzozowski@student.mini.pw.edu.pl (Ł.B.); wojtekkretowicz@gmail.com (W.K.)

**Keywords:** rammed earth, cement stabilized rammed earth, multivariate regression, random forest, artificial inteligence, features importance ranking

## Abstract

Cement-stabilized rammed earth (CSRE) is a sustainable construction material. The use of it allows for economizing on the cost of a structure. These two properties of CSRE are based on the fact that the soil used for the rammed mixture is usually dug close to the construction site, so it has random characteristics. That is the reason for the lack of widely accepted prescriptions for CSRE mixture, which could ascertain high enough compressive strength. Therefore, assessing which components of CSRE have the highest impact on its compressive strength becomes an important issue. There are three machine learning regression tools, i.e., artificial neural networks, decision tree, and random forest, used for predicting the compressive strength based on the relative content of CSRE composites (clay, silt, sand, gravel, cement, and water content). The database consisted of 434 samples of CSRE, which were prepared and crushed for testing purposes. Relatively low prediction errors of aforementioned models allowed for the use of explainable artificial intelligence tools (drop-out loss, mean squared error reduction, accumulated local effect) to rank the influence of the ingredients on the dependent variable—the compressive strength. Consistent results from all above-mentioned methods are discussed and compared to some statistical analysis of selected features. This innovative approach, helpful in designing the construction material is a solid base for reliable conclusions.

## 1. Introduction

Rammed earth is a sustainable building material; its components include inorganic soil, water, and a stabilizer. The soil is often obtained directly from the construction site, and the stabilizer is usually Portland cement [1,2]. These ingredients are mixed in an air-dry state, and then water is added. The wet, loose soil–cement mixture is laid in the formwork and then dynamically compacted in layers. Due to the use of local soil, the material is a sustainable alternative to modern ecological construction [3].

**Figure 1:**
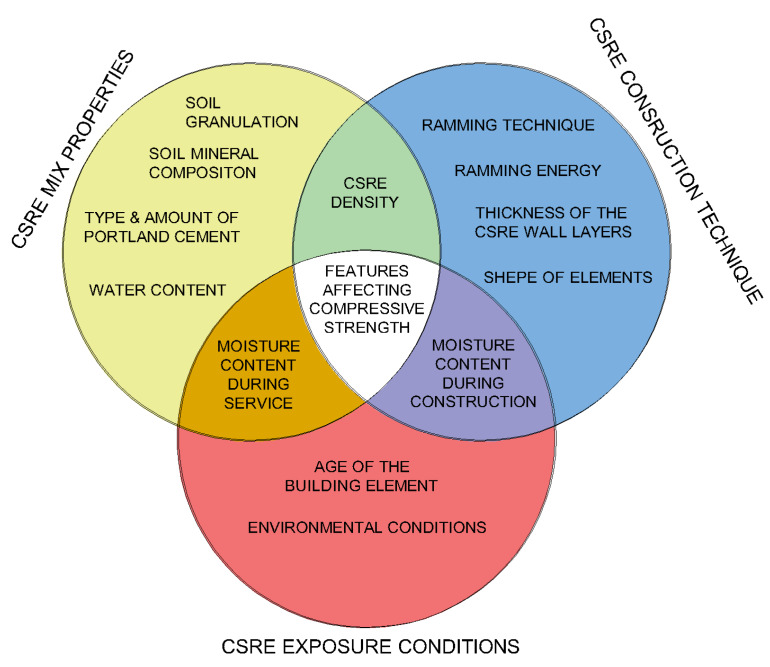
Proposed features affecting the compressive strength and durability of CSRE.

According to Eurocode 0 [4], every structure should be designed in such a way as to have adequate load-bearing capacity, usability and durability. Therefore, the critical properties of a construction building material are its durability and the compressive strength. In the case of cement stabilized rammed earth (CSRE), both properties will depend on the features of the soil–cement mixture, construction technique, and exposure conditions (Figure 1) [5,6].

The technique of ramming samples often differs both on construction site and in laboratory tests [7,8,9]. This technique, as well as curing time and conditions have a significant impact on the initial and rheological properties of the hardened material [10,11,12]. The time necessary for the CSRE wall to obtain minimum properties that allow it to continue construction (including its loading) depends on the environmental conditions—temperature and relative humidity. The increase in mechanical strength is associated with the drying process, including the course of shrinkage and the cement hydration process. It is vital to ensure such curing conditions that the CSRE elements do not stratify, become covered with mold or crack.

The durability of rammed earth is mainly related to the action of water [13]. The increase of water content in rammed earth wall and erosion are correlated [13]. CSRE durability evaluation can be done through assessing changes in its compressive strength or volume under the influence of water [5], shrinkage [14], resistance to weather conditions [15,16], water erosion resistance [5,16], water absorption [17], resistance to cyclic wetting and drying [16] and frost resistance [5,18,19]. There is a minimal amount of research concerning the corrosion of reinforcement in any kind of rammed earth in the literature [20]. This construction material is generally applied without reinforcement, except the cases where it is necessary to provide additional seismic resistance or support over openings, and anchorage for roof structures [21]. Then, steel reinforcement has to be galvanized to prevent rusting, to provide lasting the structure for decades [21,22]. Except corrosion, applying reinforcement in CESRE faces the following problems: carbonation (which can depassivate steel in long time), weaker internal forces distribution (than in reinforced concrete), and displacements of steel rebar during the process of ramming. These are the possible reasons to avoid reinforcing entirely earth-rammed structures.

The mineral composition of the soil is an essential feature that also should be considered in designing structural elements made of CSRE. It will affect both durability and mechanical strength [23]. Research [12] has shown that a CSRE element with the same soil particle size distribution, humidity, and addition of cement, but with different mineral compositions, can have significantly different compressive strength. This is due to swelling minerals (smectites) found in the clay fraction, whose presence in the soil can be determined using relatively simple laboratory tests. In the smectite group, the most widespread minerals are beidellite and montmorillonite. Montmorillonite is a highly swelling mineral with high hydrophilicity. CSRE with soli containing montmorillonite significantly reduces compressive strength [12]. Beidellite also reduces it, but to a lesser extent. This is due to the lower swelling potential of this mineral compare to montmorillonite [24]. There are also minerals whose presence increases the CSRE compressive strength, i.e., kaolinite. Kaolinite, as pozzolan, can react with cement hydration products [23]. Soil suitable for rammed earth mixtures cannot contain organic matter, which quickly absorbs water; it is highly compressible and biodegradable [12].

This study is focused on assessing the influence of each CSRE mixture component on the compressive strength. It is a crucial property of construction building material [12]. The CSRE mixture components considered in this research are the content of aggregates of different particle size distribution, cement addition, and water content. The particle size distribution is a property of soil that is also difficult to determine precisely in a construction setting. Many researchers have shown that CARE from soils that do not contain swelling minerals but have different grain sizes may have a big difference in compressive strength [12,19,23]. The soil-cement mixtures’ moisture content may change unexpectedly due to changes in atmospheric conditions prevailing at the construction site. Besides, it is difficult to accurately determine the moisture content of soil obtained from a construction site or to dry it to a constant mass.

Precise optimum moisture content determination on the construction site is difficult. Optimum moisture content (OMC) is the moisture content at which the ramming soil-cement mixture will obtain maximum dry density. This feature will depend on the soil particle size distribution, which determination may prove difficult in field conditions. A similar problem will be related to maintaining a uniform mixture’s moisture content for a long time, which will be affected by, among others, atmospheric conditions, often difficult to predict.

According to the New Zealand standard, NZS 4298: 1998 [25], in rammed earth, technology should be used with a soil with a moisture content with not more than 3% higher and not more than 5% lower than the optimum moisture content. Such a wide range of moisture content seems to indicate its secondary importance in obtaining fundamental CSRE properties, which the authors decided to verify. The addition of cement is necessary due to both the material’s durability [5] and its compressive strength [5,6,10,12,19,26]. Although walls erected from soil mixtures without stabilizers may achieve compressive strength, allowing them to perform a supporting function [27], these walls are susceptible to water erosion [5,16]. The use of stabilizers may also be necessary to ensure the smooth running of the construction process. CSRE walls should have a sufficiently high early load-bearing capacity, enabling loading related to the further stages of construction works. On the other hand, for ecological reasons, there is a tendency to limit the addition of cement [28]. To sum up, the CSRE mixes should contain a cement addition in an amount that ensures that the hardened material meets the criteria regarding, among others, compressive strength. The compressive test of CSRE samples gives precise information about the compressive strength. However, they are not available for builders, who use the soil dug close to a construction site. The lack of widely accepted receipts for CSRE, as well as random character of local soils, were a reason for searching for the rules of creating the mixture. Prioritization of CSRE components is one of the pieces of advice that can be given to builders. The influence of cement content and the necessity of achieving the optimum wetness are apparent in reaching required compressive strength. If a specific soil—evaluated on site—is suitable for CSRE structure, that is the question that cannot be answered easily. The advanced tools and techniques applied in the article allowed for answering which components of CSRE influence the compressive strength the most. So, they can be the subject of the closest attention of the builders erecting structures from CSRE. Based on the high number of prepared and tested samples of CSRE, the three machine learning tools (i.e., random forest, artificial neural networks, decision trees) are applied for predicting the compressive strength (based on amounts of CSRE components). The results achieved—prediction models and predictions themselves—are then a subject of applying other explainable tools for ranking the importance of components influencing the compressive strength of this construction material.

## 2. Materials and Methods

### 2.1. Materials, Sample Preparation, and Compressive Strength Testing Method

**Figure 2:**
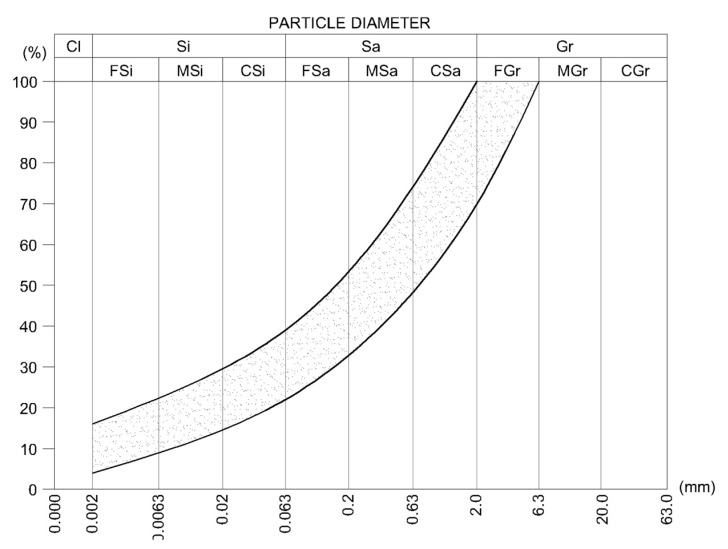
The grain size range of soils mixes used for samples preparation.

Inorganic soils for laboratory tests were prepared by mixing in a different proportion of natural 40–80% sand (0–2 mm), 0–40% gravel (2–4 mm), and 20–40% of silty clay. The sand was composed of pure quartz. Gravel fractions were composed of quartz (75% by mass) and carbonate crumbs (25% by mass). Silty clay mineral composition is shown in Table 1. The silty clay did not contain organic substances. Figure 2 shows the grain size range of the soil mixes teste. After all soil ingredients were mixed in a dry state to a homogeneous consistency different amounts (3–10%) of cement CEM I 42.5 R according to the standard EN 197-1 [29] were added and the mix was mixed again. Finally, water was added in various amounts (6–14%) and mixed again.

The CSRE samples were prepared in the cubic steel molds of dimensions 100 × 100 × 100 mm. The samples were formed in three equal layers, by freely lowering the rammer from a height of 30 cm to the surface of the moist soil–cement mixture. Each layer was rammed 20 times. The rammer had a weight of 6.5 kg and a surface of 98 × 98 mm. The samples were demolded after 24 h and then cured at a very high relative humidity of 95% (±2%) and a temperature of 20 °C (±1 °C) for 27 days. Rammed earth keeps the layered structure in the monolithic wall and similarly in the molded samples. Therefore samples were loaded in the direction in which they were rammed. The results of the compressive strength of all 434 CSRE samples, specifying their compositions, i.e., clay, silt, sand, and gravel fraction, as well as cement addition and water content, are presented in Table 2.

### 2.2. Methods of Compressive Strength Predictions

#### 2.2.1. Predictive Models

The main goal is to generate a ranking of feature importance. Therefore, the predictive models are chosen based on their fitness to feature importance calculation. It is decided to use Random Forest Regressor and Decision Tree Regressor, as they come equipped with natural feature importance measures explicitly designed for tree predictive models [30,31,32]. Neural networks (multi-layer perceptron MLP) are also chosen, as their efficient use was proven in previous research [26,33]. Eventually, we compare the models’ results with simple linear regression.

The decision tree (decision tree regressor, DTR) [32], it is a predictive model that is a binary tree. Each node corresponds to one decision, and nodes are generated to maximize the difference of samples in its’ child nodes. The final predictions are made in so-called leaves, which are nodes without child nodes. The most critical parameter is the depth of the tree [34]. The more deep-rooted tree is, the better it can fit into data. Thus, predictions are more accurate, but it is under higher risk of overfitting. Decision trees of reasonable size are simple to understand by a human. The main disadvantage is the lack of capability of predicting the target variable continuously, as the predictions are discrete. However, if the interval of values is finite, and there are many leaves, this condition is negligible [32]. The second disadvantage is the ease of overfitting. Nonetheless, it is easy to overcome it with proper hyperparameter tuning and models’ performance analysis on a cross-validated set (described further in the article).

Random forest (random forest regressor, RFR) [32], it is a predictive model composed of many decision trees – the final prediction is achieved through voting. Each decision tree is trained only on a subset of explanatory variables, thus makes the decision differently. The most critical hyperparameters are the number of trees and maximal depth [34].

Artificial neural networks, it is a machine learning model containing (in our case) fully connected layers of neurons. Each neuron in the hidden layers modifies a received signal with a proper activation function, while the neurons in the last layer modify the received values to acquire the prediction. Further information may be found in [35,36]. There are examples of successful applications of neural networks in civil engineering [26,33] and the construction material science [37,38].

Linear Regression [39], it is the simplest regression model. If a dataset X with an added column of ones, usually called the experiment matrix, has dimensions n×(m+1), that is, it has m explanatory variables (features) and n samples (observations), then the linear regression model has m+1 weights; a vector θ with dimensions (m+1)×1. Then, the prediction vector is equal:

(1) $$\hat{y}=X\theta$$

where: $\hat{y}$—vector of predicted values, X—experiment matrix, θ—vector of weights.

Vector θ can be found by minimizing mean squared error (MSE) [39,40]. The linear regression is applied with a regularization, ridge regression [41], which has added a regularization term to MSE. In that case, the value of the following expression is minimized:

(2) $$\frac{1}{n}\overset{n}{\underset{i=1}{\sum }}{\left({\hat{y}}_{i}-y_{i}\right)}^{2}+\frac{\alpha }{m}\overset{m}{\underset{i=1}{\sum }}\theta_{i}^{2}$$

where: n—number of samples, m—number of features, α—regularization coefficient, y—vector of observed values.

The regularization term is supposed to prevent overfitting [41]: the bigger α, the stronger the regularization. The main advantage of linear regression is its simplicity of interpretation. However, it often gives mediocre results and can only find linear correlations between features and the target variable [42]. The above models were then are trained on a dataset containing “Cement,” “Gravel,” “Silt,” “Moisture Content,” “Sand,” and “Clay” features to predict the samples’ compressive strength. The data are standardized before training. Many standardization techniques are known [43,44]. The calculations are based on the universal and effective technique of scaling the features (columns) so that each of them achieves mean equal to zero and variance equal to one. Such scaling yields no effect on the quality of predictions of DTR, RFR [32], and linear regression [40], while it has proven usefulness for neural networks [45].

#### 2.2.2. Performance Measures of the Predictive Models

In this section, the performance measures of the predictive models are presented, used to compare the models applied.

N-fold cross-validation [46], it is a model validation technique based on dividing the training set into 

N subsets and then iterative training of the model on N−1 of the subsets and testing its performance on the remaining one. N-fold cross-validation allows us to observe whether the model becomes overfitted, and the mean performance measures estimate the overall model performance measures as well [47].

Residuals [48]—they are the differences between predicted and observed values of the target column. The vector of residuals is given as

(3) $$r=y-\hat{y}$$

The residual analysis allows observing how well the model has been fitted.

MAE [40]—Mean Absolute Error—it is the mean of absolute values of differences between observed and predicted values. Let n be the number of observations, y be the vector of observed target values and $\hat{y}$ be the vector of predicted values. Then MAE is equal:

(4) $$\mathrm{MAE}=\frac{1}{n}\overset{n}{\underset{i=1}{\sum }}\left|y_{i}-{\hat{y}}_{i}\right|$$

MAE is a natural and straightforward measure of mean prediction error.

MSE [40] Mean Squared Error, it is an average of squared differences between observed values and predicted values.

(5) $$\mathrm{MSE}=\frac{1}{n}\overset{n}{\underset{i=1}{\sum }}{\left(y_{i}-{\hat{y}}_{i}\right)}^{2}$$

MSE, in comparison to MAE, is more vulnerable to more significant errors [49]. However, it is still a valuable measure, especially for simple models [50].

MAX—a supplemental measure of the maximal prediction error performed by the model.

(6) $$\mathrm{MAX}=\underset{i\in \left\{1,\;\ldots ,\;n\right\}}{\max}\left|y_{i}-{\hat{y}}_{i}\right|$$

R-squared [40,51] is a coefficient of determination, an adjuvant measure of the proportion of the explained to the total variation of the dataset. If its value is close to 1, this usually indicates a good fit, while the value close to 0 may indicate a bad fit. However, we use R-squared only as a supplementary measure, as its high value does not confirm the well-fitting of a model [51]. The coefficient of determination is equal:

(7) $$R^{2}=1-\frac{\sum_{i=1}^{n}{\left(y_{i}-{\hat{y}}_{i}\right)}^{2}}{\sum_{i=1}^{n}{\left(y_{i}-\bar{y}\right)}^{2}}$$

where: $\bar{y}$—the average value of y.

#### 2.2.3. Hyperparameter Tuning

To achieve a good model fit, model hyperparameter tuning is performed. It is a process of choosing the model parameters to minimize the error function. The hyperparameters are tuned through Bayesian optimization [52] process, which does not require the function to be differentiable and searches for the optimal parameters assuming that the error function is a random distribution. The algorithm iteratively calculates the error function values on a test sample randomly chosen from the previously assumed distribution, and then improves the assumed distribution based on the results (the distributions are called respectively a priori and a posteriori). Due to the increasingly better fit of the model, one may find the parameters which provide an excellent fit to the dataset relatively fast. Bayesian optimization is precisely explained in [52].

### 2.3. Feature Importance Measures

Based on predictions achieved, it can be analyzed how particular features influence the compressive strength. The following three measures are applied: drop-out loss, MSE reduction (for RFR and DTR only), and accumulated dependency.

Drop-out loss [53] is a measure of feature importance given as the difference between model performance measures before and after variable column permutation. It is a universal measure applicable to any predictive model. Let f denote the loss function of the model achieved on cross-validation sets, and x denote the variable of which the feature importance is measured. Let ${\left\{\sigma \left(x\right)\right\}}_{1}^{k}$ be a sequence of random permutations of x. The values of the loss function can be calculated in a dataset where x is replaced with $\sigma {\left(x\right)}_{i}$. Intuitively, the worse the model performance became in comparison to the initial model, the more significant is variable in the model. After repeating the operation for every 1, …, k, the new loss function values ${\hat{f}}_{1},\;\cdots ,\;{\hat{f}}_{k}$ are acquired. It can be measured then the feature importance of the variable x as:

(8) $$FI\left(x\right)=\frac{1}{k}\overset{k}{\underset{i=1}{\sum }}(f-{\hat{f}}_{i})\;$$

where: f—the value of the loss function before variable permutation, ${\hat{f}}_{i}$—the value of the loss function after i-th permutation, k—the number of permutations.

In the presented calculations, MAE is taken as the loss function and k=100. High values of drop-out loss indicate the high impact of the variable on the model performance, which then estimates the actual importance of the variable on the target, providing that the model is well fitted. In particular, the drop-out loss values may be negative, which indicates that the presence of the variable in the dataset decreases the model accuracy and that the variable needs to be removed. Moreover, when there are existing variables with high correlation measure, the values of FI tend to distribute asymmetrically between the correlated variables, thus making the measure unreliable.

Feature Importance Plot (for drop-out loss) [54]—It is a bar plot with the *y*-axis representing permuted variables, and the *x*-axis showing achieved mean drop-out loss after the variable permutations.

MSE reduction. This is a way of calculating the DTR and RFR feature importance for all independent variables [55]. For each feature, the tree is traversed, and if in the given node, the decision is based on the chosen feature, its error reduction multiplied by the number of samples that were routed to that node to its feature importance is summed. Error reduction is calculated as the difference in impurity measure of samples routed to that node minus impurities in its child nodes. MSE is taken as an impurity measure. In RFR, the resulting feature importance is a mean of feature importance of the contained trees. In the case of RFR and DTR, the achieved values are then normalized, so they sum to one. Further information may be found in [40]. However, the feature importance achieved with MSE reduction may sometimes yield biased results [56]. Therefore, the drop-out loss is considered as the primary feature importance measure.

ALE (accumulated local effect, also called an accumulated dependency) [54] describes how, on average, a particular variable changes the prediction of the model. For a given observation, it can be simulated what the model would predict if the value of the chosen feature differs (this process is called Ceteris Paribus). If an overall average of the samples and their “simulations” is taken, the Partial Dependency of the chosen feature is found as a result. However, Partial Dependency is unstable and vulnerable to linear correlations, so instead of that, a weighted average of samples and their “simulations” are taken, where weights correspond to the distance of the original samples—the bigger the distance, the lower is the weight, the less important is the sample. ALE is precisely described in [54],

The results were achieved with the use of scikit-learn package [57] for computations and model generation, and DALEX [54] with modelStudio [58] packages for drop-out loss calculation and feature importance visualization.

## 3. Results

### 3.1. Predicting model performance

Due to the strong positive linear correlation (0.95) between silt and clay, it is decided to compare feature importance and models’ audits on four variations of the dataset, as strong linear correlation may distort importance and reduce the feature importance measure’s reliability. The considered datasets are as follow:

- The dataset without any changes,
- The dataset without the clay column,
- The dataset without the silt column,
- The dataset with a column representing a sum of silt and clay values instead of two separate columns.

Sand and clay are also strongly correlated (Pearson’s correlation –0.95), but negatively. The content of these two components is balanced: the more the sand is in the sample, the more gravel can be found there. It was a reason for remaining sand and gravel as separate factors. The absolute value of all other correlations (between components) is below 0.5. The absolute value of the linear correlation between a single component and the compressive strength of CSRE is above 0.5 only for cement. It is positive and equal to 0.53, so it is a weak one. The aforementioned software allows for Bayesian optimization of DTR and RFR. The best depth of the trees is found 9 for DTR, and 20 for RFR. After several tries where the lowest MSE error was searched, the MLP regreessor architecture was set to two hidden layers (with 100 neurons in each of them) and ReLU activation function [57]. The quality of predictions is measured on cross-validated datasets with seven subsets. It is an appropriate number of folds for datasets having this number (434) of observations [46]. 

As one can observe (Table 3), the linear regression yields much worse results than the remaining models. All of them (MLP, DTR, RFR) attain very similar performance measures’ values.

**Figure 3:**
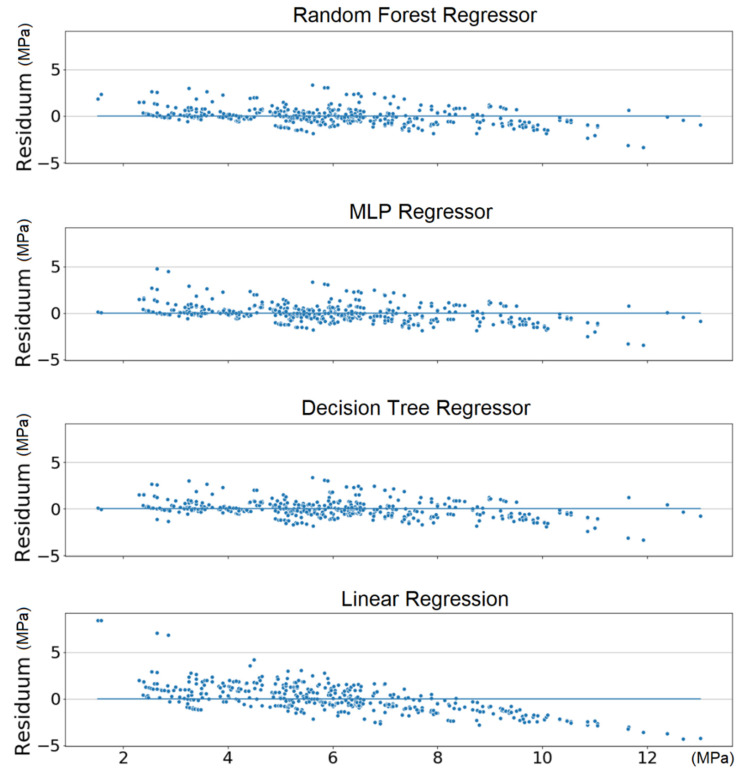
Comparison of residuals of the predictive models.

Besides the fact that the linear regression model gives much worse predictions of the compressive strength, the minimal differences between MAE values between different datasets can be observed, as well. Therefore, the following results are presented for the dataset with a column representing the sum of silt and clay, as the aforementioned differences are negligible. Residuals (presented in Figure 3) of linear regression are much higher than of the other models and they are significantly unevenly distributed. It can be concluded that the linear regression model is insufficient in predicting the compressing strength, and we discard it in the following computations.

**Figure 4:**
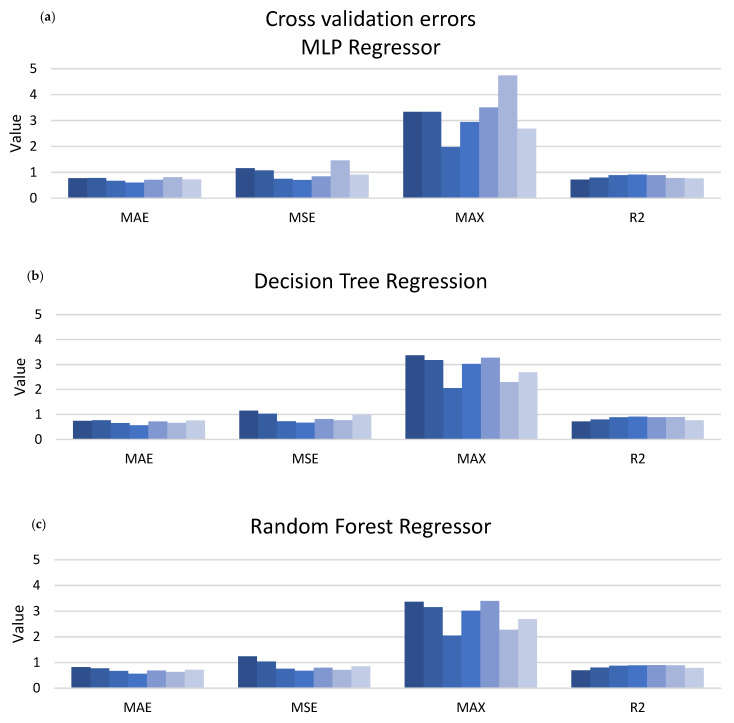
Performance measures’ values of the three remaining models on the last dataset. (**a**) for MLP regressor, (**b**) for DTR, (**c**) for RFR.

The measures of MAE, MSE, MAX, and R2 are presented in Figure 4 for all seven subsets of the cross-validated datasets. Every single column corresponds to one performance measure attained on one subset. It can be observed in Figure 4 that MLP, RFR, and DTR models behave pairwise almost identically. The following measures, MAE, MSE, R2, have different units (defined in Section 2.2.2), which is the reason for omitting to present the unit of the vertical axis in Figure 4.

Results achieved on the cross-validated set indicate that the models are not overfitted. Moreover, the values of MAE and MSE are relatively low, so the models have high enough accuracy for us to use them for generating the feature importance ranking.

### 3.2. Feature Importance Calculations

**Figure 5:**
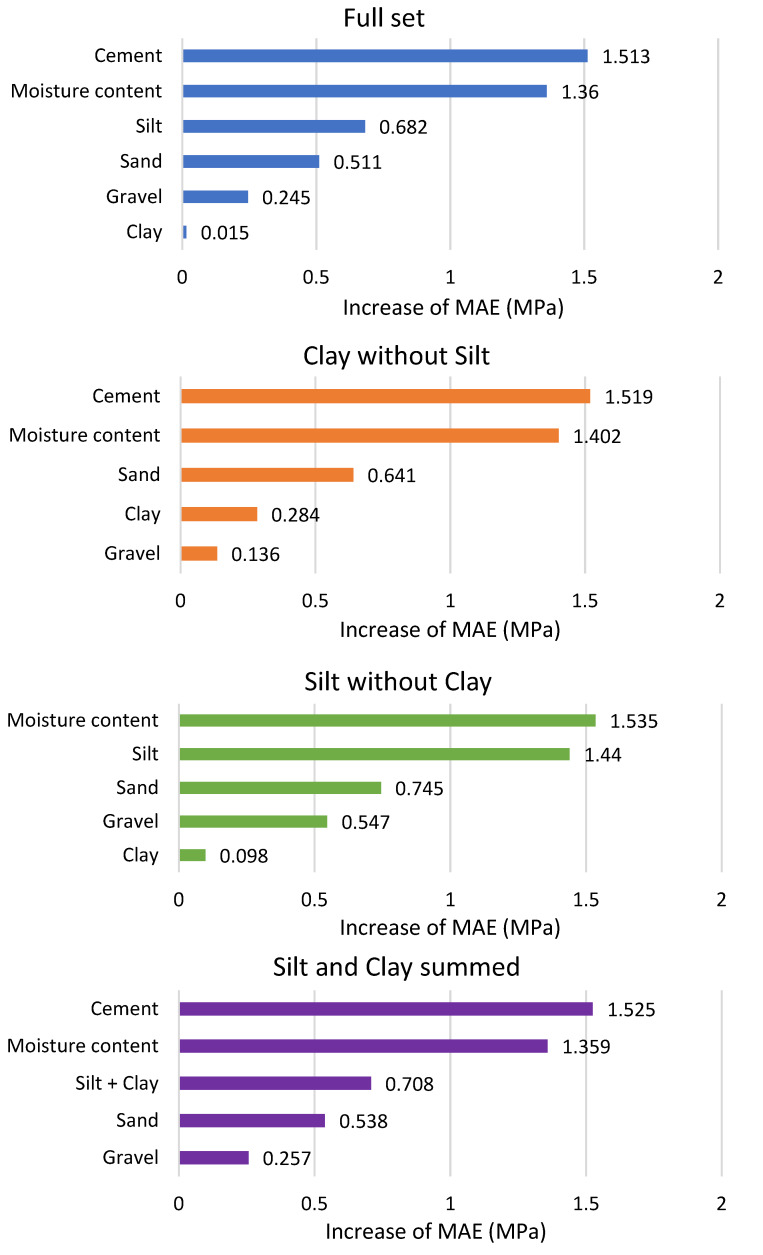
Feature importance plots from drop-out loss based on the DTR model and different datasets.

As previously mentioned, there is a strong, positive correlation between the content of silt and clay in CSRE samples. Feature importance calculated for all features (with the use of DTR model) has ranked the influence (on the compressive strength) of clay on the third place and the influence of gravel on the last place (the minimal influence). However, when the silt is removed from the database, clay becomes more influential (more than gravel). It can be observed in Figure 5 i.e., in feature importance plot. Considering that, and the strong, positive correlation between silt and clay, it was decided to analyze the summed content of silt and clay in CSRE mixture. Another important reason for making calculations for the summed content of silt and clay is that these two fractions of aggregates often appear jointly [19].

**Figure 6:**
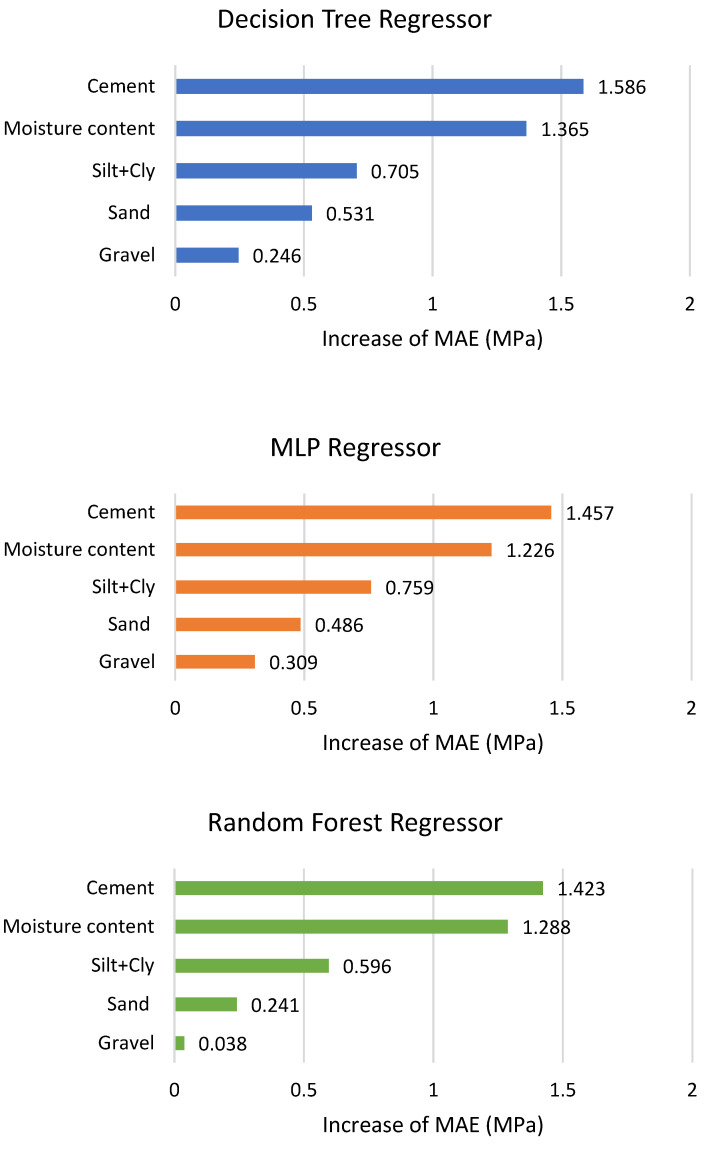
Feature importance plots from drop-out loss based on DTR, MLP regressor, and RFR (silt and clay jointly considered).

Once the decision about considering the joined impact of silt and clay on the compressive strength of CSRE is made, the cause of possible distortions of the result achieved is lowered. The calculations of drop-out loss could be made for all three models. Based on these calculations, feature importance plots are presented in Figure 6.

**Figure 7:**
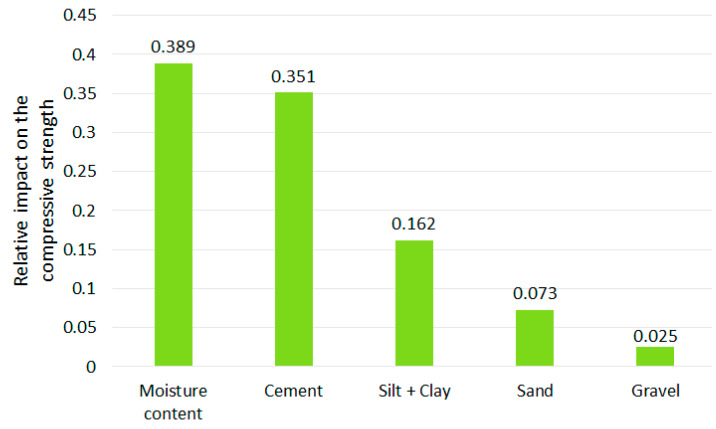
Features’ importance ranking from MSE reduction applied for RFR.

**Figure 8:**
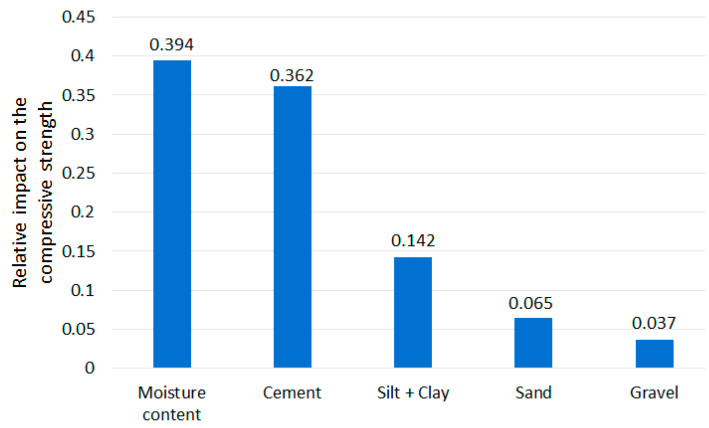
Features’ importance ranking from MSE reduction applied for DTR.

As it was described in Section 2.3, applying MSE reduction to find the features’ ranking is possible in RFR and DTR tools. The results are presented in Figure 7 and Figure 8. The scale of the vertical axis has no unit; it is comparative. The joint influence of all CSRE components sums to 1.

The results achieved based on drop-out loss and MSE reduction are collected in the following Table 4.

The results from the accumulated local effect (ALE, called also accumulated dependency) tool do not create a ranking of features influencing the compressive strength of CSRE themselves. The ranking can be created based on ALE. It was decided to utilize results achieved from accumulated dependency to verify and discuss the rankings from drop-out loss and MSE reduction tools. It is presented in the following section.

## 4. Discussion

The content of cement, as well as the water to cement ratio, are crucial in achieving required compressive strength for a traditional cement concrete [59,60]. Rankings made through drop-out loss (applied for MLP, RFR, and DTR) and MSE reduction (based on RFR and DTR) present a similar importance sequence of CSRE mixture components. The scales (i.e., their units) of these two rankings are different, so the numbers cannot be compared (i.e., the strength of the impact of the same component taken from different rankings). The difference in the most influential CSRE ingredient on the compressive strength of this structural construction material is illusionary. MSE reduction ranked the water content at the first place and cement content at the second one (the same for DTR and RFR). Drop-out loss for all three tools ranked in opposite to these two components. This is a consistent result. The processes of designing and preparing CSRE mixture usually end with matching the water addition to the amount of cement used, and to the amount of water already included in the aggregates [1].

**Figure 9:**
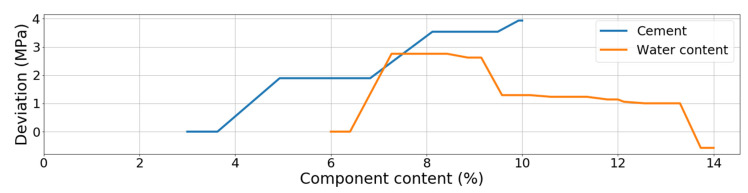
ALE plots for cement and water content based on DTR.

Based on these two sets of ranking, it can be said that the amount of cement and the amount of water (matched to the content of cement) plays a crucial role in achieving the high compressive strength of CSRE. Accumulated dependency plots are calculated for DTR. They are assumed to analyze the influence of changes of only one feature (CSRE component) at one time (assuming the other features remain unchanged) on the output (compressive strength). Reading the ALE plot concerning the content of cement (see Figure 9) requires assuming the initial content of cement in the theoretical sample e.g., 6%. Then, it can be noticed that if the cement content is lowered to 4%, the unknown initial compressive strength will be lowered by approximately 1.5 MPa. Oppositely, when the cement content is increased up to 7.5%, the unknown initial compressive strength will rise by approximately 0.8 MPa.

The more the cement content changes, the more the compressive strength of CSRE changes. This close to linear, steadily increasing dependency cannot be observed for water content. Lowering water content from 9.5% to 8.0% increases the output by approximately 1 MPa but lowering it more, down to 6.5% decreases the compressive strength by 1.5 MPa. This calculated phenomenon is confirmed by practical engineering [61,62,63]. It is due to the fact that a certain amount of cement requires an optimum amount of water, and the changes of the compressive strength observed on the plot (Figure 9) are also significant. The result from ALE can be transformed into ranking, based on the maximum range of the output changes (caused by changes of a component content). In the case of cement, the maximum change is 4 MPa. In the case of moisture content, close to 4 MPa, but less than the influence of cement content. So, applying the three explaining tools to the DTR model, the one-time (based on MSE reduction) moisture content is found as the most influential on the compressive strength, and two-times cement as the more important feature (based on drop-out loss and ALE). This kind of voting can be crucial for analyzing other phenomena. In the case of ranking of CSRE components, the results are consistent. The cement and moisture content should be matched during production process (to maximize the compressive strength).

**Figure 10:**
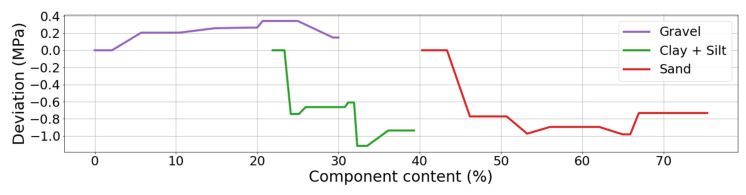
ALE plots for clay+silt, sand and gravel content based on DTR.

Both methods of ranking the features (drop-out loss and MSE reduction) placed silt and clay on the third position, just after cement and water content. It can be read in accumulated dependency plot (Figure 10) that the more the silt and clay content increases, the more the compressive strength decreases. The maximum range of MPa changes (caused by the changes of clay and silt content) is approximately 1 MPa, which is much lower than for cement and water. When preparing ALE plots for sand and gravel (Figure 10) it can be said that changes in its content in the CSRE mixture almost do not affect the compressive strength. The maximum range of compressive strength changes, calculated for sand is slightly lower than for silt and clay. The influence of gravel could be practically neglected. The ranking based on the analysis of results from accumulated dependency can be created. Starting from the most influential feature it is cement, moisture content, clay and silt, sand, gravel. ALE is based on the tested samples. The step character (step-shelf-step) of the curves (presented in Figure 9 and Figure 10) arises from the fact that changes of components’ content in CSRE mixtures were not continuous—also made in steps. In analysis with a much higher number of cases, where features are continuous, the reasoning based on the width of horizontal “shelves” of ALE curves can be done. It is not possible in the presented case. The reasoning based on ALE plots is inspired [64,65,66].

The proposed methods of ranking the influence of descriptors (amounts of CSRE components) on a dependent value (the compressive strength) through explainable artificial intelligence tools can be applied only if there is enough samples tested (several dozen are too little; 434 samples are just on the border). The next limitation concerns ALE and drop-out loss tools. Permutating the values of one feature or shifting values of one feature, and remaining values of other features unchanged, the above-mentioned models deal with cases where the sum of components does not equal 100%. Despite the limitations of the methods, and even though the tests were carried out only for samples made of aggregates presented in Table 2 and Figure 2, the clear (consistent with all applied in the article methods) ranking of the features influencing CSRE compressive strength can be created:

1. cement and water content
2. silt and clay content
3. sand content
4. gravel content

**Figure 11:**
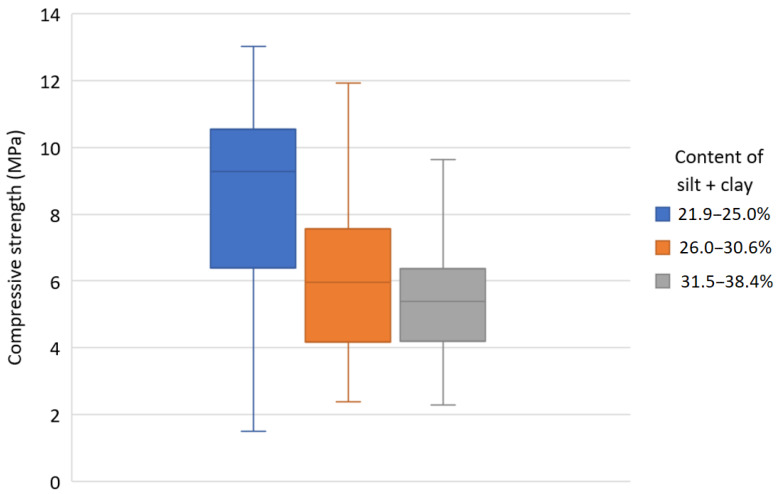
Quartiles of the CSRE compressive strength for three ranges of silt and clay content.

Figure 11 confirms the rule that the more silt and clay is in a CSRE mixture, then its compressive strength decreases. The presented box graphs are based on all 434 samples tested (with different all components of mixtures, as presented in Table 2). The quartiles of the compressive strength are presented for three ranges of clay and silt content (covering its full range in tested samples).

When the cement content is also considered (as a differentiating factor), the effect of the negative influence of clay and silt on the compressive strength can be observed too. Table 5 is prepared for samples where the water to cement ratio is from 0.67 to 1.67. These samples are divided into three groups according to the amount of cement added. Then, the median value of the compressive strength is found for each group. For every group, the average content of clay and silt is lower for the samples with the compressive strength above the median value, than for samples where the compressive strength is below the median value.

These findings have significant practical meaning for assessing the quality of dug, on-site soil: the component of cement stabilized rammed earth. Much more important for achieving higher compressive strength is the low content of clay and silt, then the proportion of sand and gravel in the soil on site. When the low content of clay and silt is checked, even 6% of cement added can lead to the compressive strength above 5.5 MPa.

Based on the prepared calculation, the tools used for them can also be assessed. There is no meaningful difference in the prediction accuracy between DTR, RFR, and MLP regression models. A numerous number of samples can be analyzed with their use. However, it is important to remember, that MSE reduction is not a universal method of preparing features’ ranking based on machine learning. It can be applied for tree-based tools as DTR and RFR in the article. Drop-out loss and accumulated dependency (ALE) methods can be applied for every machine learning predictor, but result from ALE is not the explicit ranking. The ranking is created based on results from ALE. Nevertheless, an accumulated dependency is very helpful, in the article, in comparing rankings achieved through drop-out loss and MSE reduction, as well as, for explaining not obvious consistency of these rankings. The multivariate linear regression model is applied only as a background, legitimating the use of machine learning tools for the compressive strength predictions. From the set of machine learning tools applied, only one from the pair DTR and RFR could be omitted without lowering the quality of results and conclusions.

## 5. Conclusions

The three machine learning tools, i.e., DTR, MLP, and RFR, are applied to predict the compressive strength of CSRE. The error of predictions (MAE) around 11% of the observed values allowed the use of explainable tools as drop-out loss, MSE reduction, and accumulated dependency. Considering the required correlation between cement and water content and considering silt and clay jointly, the calculated rankings are consistent. CSRE components are ranked according to their influence on the compressive strength. The sequence, starting from the most influential component, is cement and water (considered jointly), clay and silt (also considered jointly), sand, and gravel. The positive influence of cement and water is evident for builders (the higher content of cement, the higher the compressive strength). The unexpected, strong impact of silt and clay (when compared to the influence of sand and gravel) is found with the proposed procedures. The negative influence of clay and silt is more important than the content of sand or gravel.

These findings are valid for the samples created (their details can be found in Section 2). Nevertheless, as it is presented in Section 4, the limitations of artificial intelligence (AI) tools are not a barrier in successfully applying them in CSRE (composite material) examination and design. The rule concerning the meaningful negative impact of clay and silt on the CSRE compressive strength can be applied within the range of aggregate compositions presented in Figure 2. The usefulness of explainable artificial intelligence tools for such analysis is proved in the article as well, so the method can be applied for other, broader sets of data concerning crush tests of composite construction materials.

It is recommended to use at least two machine learning tools for predicting the dependent variable in this kind of analysis, based on varying algorithms (as, e.g., MLP and RFR can vary). The features’ ranking tools (MSE reduction, drop-out loss) may produce slightly different rankings for different prediction models, or may not be applied for all prediction models (as MSE reduction for MLP cannot be applied). Accumulated dependency was not designed specifically as a ranking tool, but it can help explain and verify any potential differences between the rankings, as described in the article.

## Figures and Tables

**Table 1:** Mineral and chemical composition of silty clay used in examined soil mixtures [5].

Mineral Composition [%]									
	Clay Minerals	Including:			Goethite	Siderite	Carbonates	Organic Substance	Quartz and Other
		Beidellite	Kaolinite	Illite					
Weight content in silty clay (%)	43.7	8.9	8.6	26.2	-	6.0	-	0	50.3

**Table 2:** Composition and the compressive strength of 434 tested samples—basic statistics.

	Silt	Clay	Sand	Gravel	Cement	Moisture Content	Compressive Strength (MPa)
Min	7.0%	14.9%	40.3%	0.0%	3.0%	6.0%	1.52
Max	14.0%	25.3%	75.4%	30.0%	10.0%	14.0%	13.01
Mean	10.4%	20.4%	52.6%	16.5%	7.6%	9.9%	5.99
Median	10.5%	20.1%	49.4%	20.0%	9.0%	10.0%	5.85
Standard deviation	1.69%	2.21%	11.33%	11.74%	2.13%	1.72%	2.21

**Table 3:** Performance measures’ values of the predictive models.

Linear Regression				
Error Measure	Silt and Clay	Without Clay	Without Silt	Summed
MAE	1.17	1.17	1.17	1.17
MSE	2.41	2.41	2.41	2.41
MAX	5.31	5.31	5.31	5.31
R2	0.49	0.49	0.49	0.49
**Decision tree**				
Error measure	Silt and Clay	Without Clay	Without Silt	Summed
MAE	0.68	0.68	0.68	0.68
MSE	0.86	0.85	0.85	0.85
MAX	2.83	2.83	2.83	2.83
R2	0.81	0.81	0.81	0.81
**Neural networks**				
Error measure	Silt and Clay	Without Clay	Without Silt	Summed
MAE	0.73	0.71	0.70	0.70
MSE	1.04	1.00	1.01	0.96
MAX	3.24	3.22	3.30	3.19
R2	0.78	0.78	0.78	0.79
**Random forest**				
Error measure	Silt and Clay	Without Clay	Without Silt	Summed
MAE	0.67	0.67	0.67	0.67
MSE	0.85	0.85	0.84	0.85
MAX	2.83	2.83	2.83	2.83
R2	0.81	0.81	0.81	0.81

**Table 4:** Summary of the five rankings (the most important component is at the top of each of the lists).

Drop-Out Loss for DTR	Drop-Out Loss for MLP	Drop-Out Loss for RFR	MSE Reduction for DTR	MSE Reduction for RFR
Cement	Cement	Cement	Moisture	Moisture
Moisture	Moisture	Moisture	Cement	Cement
Silt + Clay	Silt + Clay	Silt + Clay	Silt + Clay	Silt + Clay
Sand	Sand	Sand	Sand	Sand
Gravel	Gravel	Gravel	Gravel	Gravel

**Table 5:** The content of clay+silt for different groups of samples.

The Cement Content %	Number of Samples	The Median Value of Compressive Strength Mpa	The Mean Content of Clay + Silt %	
			For Samples with the Compressive Strength above the Median Value	For Samples with the Compressive Strength Below the Median Value
6	108	5.577	29.6%	31.5%
9	210	6.533	29.8%	32.4%
10	46	5.900	29.8%	30.9%
